# Production and Properties of Microbial Polyhydroxyalkanoates Synthesized from Hydrolysates of Jerusalem Artichoke Tubers and Vegetative Biomass

**DOI:** 10.3390/polym14010132

**Published:** 2021-12-30

**Authors:** Tatiana G. Volova, Evgeniy G. Kiselev, Alexey V. Demidenko, Natalia O. Zhila, Ivan V. Nemtsev, Anna V. Lukyanenko

**Affiliations:** 1 Basic Department of Biotechnology, School of Fundamental Biology and Biotechnology, Siberian Federal University, 660041 Krasnoyarsk, Russia; volova45@mail.ru (T.G.V.); evgeniygek@gmail.com (E.G.K.); kraysolnca@mail.ru (A.V.D.); ivan_nemtsev@mail.ru (I.V.N.); lav@iph.krasn.ru (A.V.L.); 2 Institute of Biophysics SB RAS, Federal Research Center “Krasnoyarsk Science Center SB RAS”, 660036 Krasnoyarsk, Russia; 3 L.V. Kirensky Institute of Physics SB RAS, Federal Research Center “Krasnoyarsk Science Center SB RAS”, 660036 Krasnoyarsk, Russia; 4 Federal Research Center “Krasnoyarsk Science Center of the Siberian Branch of the Russian Academy of Sciences”, 660036 Krasnoyarsk, Russia

**Keywords:** Jerusalem artichoke hydrolysates, PHA synthesis, productivity, polyhydroxyalkanoates

## Abstract

One of the major challenges in PHA biotechnology is optimization of biotechnological processes of the entire synthesis, mainly by using new inexpensive carbon substrates. A promising substrate for PHA synthesis may be the sugars extracted from the Jerusalem artichoke. In the present study, hydrolysates of Jerusalem artichoke (JA) tubers and vegetative biomass were produced and used as carbon substrate for PHA synthesis. The hydrolysis procedure (the combination of aqueous extraction and acid hydrolysis, process temperature and duration) influenced the content of reducing substances (RS), monosaccharide contents, and the fructose/glucose ratio. All types of hydrolysates tested as substrates for cultivation of three strains—*C. necator* B-10646 and *R. eutropha* B 5786 and B 8562—were suitable for PHA synthesis, producing different biomass concentrations and polymer contents. The most productive process, conducted in 12-L fermenters, was achieved on hydrolysates of JA tubers (X = 66.9 g/L, 82% PHA) and vegetative biomass (55.1 g/L and 62% PHA) produced by aqueous extraction of sugars at 80 °C followed by acid hydrolysis at 60 °C, using the most productive strain, *C. necator* B-10646. The effects of JA hydrolysates on physicochemical properties of PHAs were studied for the first time. P(3HB) specimens synthesized from the JA hydrolysates, regardless of the source (tubers or vegetative biomass), hydrolysis conditions, and PHA producing strain employed, exhibited the 100–120 °C difference between the T_melt_ and T_degr_, prevailing of the crystalline phase over the amorphous one (C_x_ between 69 and 75%), and variations in weight average molecular weight (409–480) kDa. Supplementation of the culture medium of *C. necator* B-10646 grown on JA hydrolysates with potassium valerate and ε-caprolactone resulted in the synthesis of P(3HB-co-3HV) and P(3HB-co-4HB) copolymers that had decreased degrees of crystallinity and molecular weights, which influenced the porosity and surface roughness of polymer films prepared from them. The study shows that JA hydrolysates used as carbon source enabled productive synthesis of PHAs, comparable to synthesis from pure sugars. The next step is to scale up PHA synthesis from JA hydrolysates and conduct the feasibility study. The present study contributes to the solution of the critical problem of PHA biotechnology—finding widely available and inexpensive substrates.

## 1. Introduction

One of the main environmental problems today is plastic pollution. Petroleum-based polymers are not biodegradable, and most of the plastic waste is released into the environment or collected in landfills. Conventional plastics are resistant to microbial degradation, accumulating in the environment and food chains [1].

The development of degradable polymer materials, which are able to join material cycling in the biosphere, is among the top-rated critical technologies of the 21st century, putting strong emphasis on research into biodegradable plastics as an alternative to non-degradable synthetic materials [2,3]. Biodegradable polymer materials were first produced in the 1980s [4]. In contrast to synthetic plastics, biopolymers are produced from renewable sources such as corn, sugar, and potatoes. Bioplastics are not toxic, and they are quickly degraded in the environment [5]. Despite the environmental safety of bioplastics, they constitute only a small percentage of the total production of plastics. The data presented in the Global Bioplastics Report 2020 show that in 2019, the global production of plastics reached 368 million tons, and bioplastics constituted only about 1% of them [6].

Natural biodegradable biopolymers are represented by macromolecular compounds such as polysaccharides and proteins. Synthesis of biodegradable synthetic polymers including poly(butylene succinate) (PBS), poly(butylene adipate-co-terephthalate) (PBAT), etc. remains in the field of consumption of nonrenewable petrochemicals. Degradable synthetic polymers such as poly(lactic acid) (PLA), poly(glycolic acid) (PGA), and copolymers thereof, which are manufactured on a commercial scale, have been increasingly used lately.

Polymers of hydroxyalkanoic acids, polyhydroxyalkanoates (PHAs), are true products of biotechnology, so-called “green plastics”. This is a family of polymers differing in their chemical composition and produced from various carbon sources, including waste materials [7,8,9,10,11,12,13]. Being UV resistant, non-hydrolyzed in liquids, thermoplastic, biodegradable, and highly biocompatible, PHAs are promising materials of the 21st century and major competitors not only to the conventional polymers, but also to well-known biodegradable plastics (polylactide, polyethylene terephthalate, polyamides, etc.). Owing to their unique properties, biopolymers can be used in various applications, from biodegradable packaging to biocompatible medical devices and tissue engineering [14,15,16,17,18,19,20,21,22]. Commercial-scale PHA production and wider application are hindered by the cost of these polymers, which remains high [23]. Thus, the main challenge PHA biotechnology is facing is optimization of synthesis processes, primarily by using new and widely available carbon sources.

The most common substrate in biotechnology is sugars. Sugar-containing industrial and agricultural wastes and hydrolysates of various plant materials are an inexhaustible renewable substrate resource for microbiological manufacture of biotechnological target products including PHAs [24]. Research has been carried out to study hydrolysates of various plants as potential sugar-containing substrates for PHA production: wood [25], sunflower stalks [26], *Miscanthus* biomass [27], spent coffee grounds [28], oil palm [29], straw and husk of cereal grain crops [30,31,32], sugar beet and sugar cane cakes and peels [33], hydrolysate of noxious weed water hyacinth biomass [34], hydrolysate of wheat waste biomass [35], etc.

PHAs can also be produced from the pretreated lignocellulose. Preliminary aqueous extraction is used to extract mono-, oligo-, and easy-to-hydrolyze polysaccharides; this treatment is performed to prevent oxidation of this part of the total carbohydrates during the subsequent acid hydrolysis. A disadvantage of this method is a rather low sugar yield, leading to highly diluted solutions. If no pretreatment is conducted, acid hydrolysis causes fructose oxidation to toxic furans, negatively affecting bacterial cells in the biotechnological process. Adverse effects of the impurities contained in acid hydrolysates were reported by a number of authors [25,27,30]. The use of aqueous extracts of lignocellulose stock material in PHA biosynthesis was mentioned in a study by Kulkarni (2015) [33].

A promising but insufficiently studied substrate for PHA synthesis may be the sugars extracted from the Jerusalem artichoke. The Jerusalem artichoke (JA), also called sunroot or wild sunflower (*Heliánthus tuberósus*), is an annual plant of the Asteraceae family, with high crop yields—up to 40–70 t/ha for tubers. JA can grow on different soils and in different climates, from dry deserts to frost-prone regions, unsuitable for growing food crops such as wheat, rice, and potato [36]. The tubers of JA contain a unique carbohydrate group based on fructose and its polymers, whose higher homologue is inulin [37,38,39]. Inulin is enzymatically hydrolyzed to fructose and glucose due to the combined action of inulinase and invertase [40]. The inulin content of the tubers constitutes 70–80% of the total dry weight [41,42]. Inulin mainly consists of fructose (85–95%), and fructose content of JA varies depending on the harvesting dates, storage duration, etc., as it is the product of inulin metabolism in JA roots and tubers [39]. JA tubers also contain large amounts of inulids, which are depolymerized to inulin by the enzymes. In addition to inulin, the tubers contain free sugars, proteins, and minerals [43].

JA, as well as chicory, is a source for inulin production. To extract inulin and other products (ethanol, yeast biomass, food enzymes, high-fructose syrups, and lipids), JA tubers or, much less frequently, vegetative biomass are subjected to chemical or enzymatic hydrolysis [44,45,46,47,48,49,50]. However, PHA synthesis from JA sugars remains insufficiently studied, especially because many of the PHA producers, including strains of *Cupriavidus necator*—a generally recognized high-productivity PHA producer from various sources, have no inulinase—a hydrolytic enzyme needed to convert JA inulin into substrates available to bacterial cells, fructose and glucose [39].

There are special approaches and processes consisting of several stages, e.g., hydrolysis followed by fermentation or the use of several strains differing in their metabolic potentials, which show that JA sugars can be used as carbon source for the synthesis of PHAs, chiefly the homopolymer of 3-hydroxybutyric acid, P(3HB). In a study by Koutinas et al. (2013), ground JA tubers were first used as substrate in solid state fermentation of *Aspergillus awamori* to produce the enzymes invertase, protease, and inulinase [36]. Then, remaining solids containing crude enzymes were placed into the aqueous solution of ground JA tubers for the hydrolysis of sugar-containing and nitrogen components into fructose, glucose, peptides, and amino acids, respectively. The crude hydrolysates containing hexoses available to bacterial cells were used as substrate for *Cupriavidus necator* DSM 4058 cultivation, resulting in P(3HB) content reaching 51.9%. In a study by Corrado et al. (2021), PHAs were synthesized by *C. necator* DSM 428 on the medium containing inulin and a fungal inulinase mixture; the study demonstrated effective use of the fungal inulinase mixture in a process for polyhydroxyalkanoate (PHA) production by *Cupriavidus necator* from inulin [39]. P(3HB) synthesis was carried out using hydrolysates of chicory roots—a more commonly used inulin source [51]. The authors of that study cultivated three *Cupriavidus strains* (*C. necator* DSM 428, *C. necator* DSM 531, and *C. necator* DSM 545) and showed that hydrolysates of chicory roots were a suitable substrate for P(3HB) synthesis, but polymer contents and biomass concentrations differed depending on the strain used. The search of the available literature shows that the use of acid (not enzymatic) hydrolysates of JA was only studied in exploratory experiments conducted by researchers of the Institute of Biophysics SB RAS in cooperation with the Siberian Technological University (Krasnoyarsk, Russia) [52,53]. Those experiments demonstrated that acid hydrolysates of the JA vegetative biomass were a suitable substrate for P(3HB) synthesis

The present study, for the first time, reports integrated investigations of hydrolysates prepared from JA tubers and vegetative biomass and results of employing them as carbon source for PHA synthesis. Taking into account the effect of the carbon source on polymer composition, a special study was performed to investigate the chemical composition and properties of the PHAs synthesized by *Cupriavidus necator* from JA hydrolysates prepared using different procedures.

## 2. Materials and Methods

### 2.1. Materials

All simple salts (purity 99%) were purchased from “AlfaKhim” (Saint-Petersburg, Russia). Fructose (purity 99%) and glucose (purity 98%) were purchased from Panreac (Barcelona, Spain) and ZAO Khimreaktivsnab (Ufa, Russia), respectively. ε-caprolactone (purity ≥ 99%) and valeric acid (purity ≥ 99%) were purchased from Thermo Fisher Scientific (Kandal, Germany) and Sigma-Aldrich (Saint Louis, MO, USA), respectively. All solvents were chemically pure and purchased from “Ekos-1” (Staraya Kupavna, Russia). Jerusalem artichoke cv. Interes was grown in the garden plot at the city of Krasnoyarsk (Siberia, Russia) in 2020.

The strains used in the present study were from the collection of the authors of the paper (collection of the Laboratory of Chemoautotrophic Biosynthesis at the Institute of Biophysics SB RAS), registered in the Russian National Collection of Industrial Microorganisms: *Ralstonia eutropha* B 5786 [54], metabolizing only fructose for growth; *R. eutropha* B 8562—a glucose-assimilating mutant strain [55,56]; *Cupriavidus necator* B-10646, utilizing glucose and fructose, tolerant to various precursor substrates, and synthesizing PHAs with different composition [57]. Those strains were chosen because bacteria of the *Cupriavidus* (formerly known as *Wautersia*, *Ralstonia*, *Alcaligenes*, *Hydrogenomonas*) genus are regarded as the most promising PHA producers [58]. In addition to being capable of autotrophic growth and PHA synthesis from mixtures of carbon dioxide and hydrogen [59,60,61,62,63], bacteria of this genus have broad organotrophic potential and are able to synthesize PHAs at high yields from substrates differing in the degree of reduction and energy content.

### 2.2. Production of Sugar-Containing Substrates from Jerusalem Artichoke

Sugar-containing substrates were derived from the tubers and vegetative biomass (stems and leaves) of the Jerusalem artichoke cv. Interes. To carry out hydrolysis, ground tubers or vegetative biomass were mixed with hot water in the ratio 1:7, taking into account the initial moisture content of the plant material. The choice of the temperature of hydrolysis was based on the well-known data indicating that the highest temperature of sugar hydrolysis in the acidic medium must not exceed 70–80 °C to avoid subsequent reactions of monosaccharides and formation of HMF (5-hydroxymethylfurfural), D-fructose dianhydrides, and humic substances, which deactivate catalysts and “contaminate” the solutions of monosaccharides, thus complicating the fermentation involving microorganisms sensitive to such toxic compounds [64,65,66,67].

**Figure 1:**
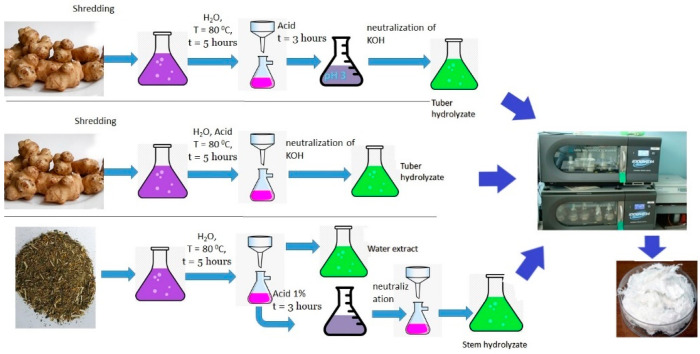
The scheme of hydrolysate production from JA tubers and vegetative part used as substrate for PHA synthesis by the bacteria *C. necator* B-10646, *R. eutropha* B5786 and 8562.

As sugars had been successfully extracted by acid hydrolysis of JA [68,69], and hydrolysates had been employed to synthesize P(3HB) [52,53], in the present study, sugars were extracted using two procedures. The first procedure was to use a sequential two-phase scheme: in Phase 1, aqueous extraction of sugars was performed at 80 °C; in Phase 2, the resulting oligosaccharide extract was subjected to acid hydrolysis at 60 °C. The second procedure was to perform sugar extraction and hydrolysis in a weakly acidic medium simultaneously at 80 °C (Figure 1).

The JA tubers were washed and ground with a meat grinder (the moisture content of the paste was 74%). Under the first procedure, a sample of the ground JA tubers (100 g ODW) was soaked in water (tubers: water = 1:7) at a temperature of 80 °C for 4–5 h. The resulting extract was filtered, and pH was adjusted to 3 using sulfuric acid (“Ekos-1”, Staraya Kupavna, Russia; chemically pure). The post-extraction residue was washed with 500 mL water. The wash water was combined with the extract. Then, the extract oligosaccharides were subjected to hydrolysis under heating to 60 °C for 3 h. Under the second procedure, the first step was the same as in the first procedure: a sample of the ground JA tubers (100 g ODW) was soaked in water (tubers: water = 1:7), with pH adjusted to 3, and maintained at a temperature of 80 °C for 4–5 h. Then, the resulting hydrolysate was passed through filter paper, and the residue was washed with distilled water. The wash water was combined with the hydrolysate. In contrast to the first procedure, extraction and hydrolysis of oligosaccharides occurred simultaneously, and the fiber of the tubers was partially hydrolyzed [68]. Acid hydrolysates of JA tubers were neutralized with 0.5 N KOH (ZAO Khimreaktivsnab, Ufa, Russia; chemically pure), to pH 6.5–7.0 and evaporated to one half the volume) (Figure 1).

The vegetative part of JA plants (stems and leaves) with moisture content of 10–12% was ground with an AS200 grinding machine with a 0.1 mm sieve. Hydrolysis of the vegetative biomass was also carried out using two procedures. The first procedure was the same as the first procedure of tuber hydrolysis (described above). Under the second procedure, the ground JA vegetative biomass was subjected to hot water extraction. Plant biomass (25 g ODW) was soaked in water (1:7) at a temperature of 80 °C for 5 h. The resulting extract was filtered, and post-extraction residue was washed with 500 mL water. The wash water was combined with the extract. The residual vegetative biomass after extraction was hydrolyzed with 1% sulfuric acid (1:7) and maintained at 100 °C for 8 h under stirring. The resulting hydrolysate was filtered, the residue was washed with distilled water, and the wash water was combined with the hydrolysate. Then, the hydrolysate was neutralized with KOH to pH 6.5–7.0 [69] (Figure 1).

### 2.3. Composition of Jerusalem Artichoke Hydrolysates

Chemical composition of the JA hydrolysates was studied using the procedures accepted in chemistry of plant raw material [70]. Easy- and hard-to-hydrolyze polysaccharides were determined using the Kizel and Semiganovsky method. This method is based on determining the amount of monosaccharides formed from easy-to-hydrolyze polysaccharides hydrolyzed by dilute hydrochloric acid and on determining the amount of monosaccharides formed from hard-to-hydrolyze polysaccharides contained in the residual plant tissue after hydrolysis of easy-to-hydrolyze polysaccharides. Hydrolysates were analyzed to determine the content of reducing substances (RS) by the direct titration method based on oxidation of reducing substances with copper-alkaline solution (using Fehling’s solution) during boiling [71].

Hot water extraction from the JA vegetative part was performed in a flask with reflux condenser by boiling for 3 h. The amount of water-soluble substances was determined from the mass of the dry residue after evaporation of the aqueous extract (sample aliquot) [69,71]. The aqueous extract was analyzed for the reducing substances before and after inversion with sulfuric acid, for nitrogen, micro- and macronutrients, and sugars.

Concentration of fructose was determined using the resorcinol method [72]; concentration of glucose—spectrophotometrically at 490 nm by the glucose oxidase method using a Fotoglucoza kit (Impact Ltd., Moscow, Russia).

Nitrogen concentration in the culture medium was analyzed at different time points, using a photometric method, with Nessler’s reagent. To measure concentrations of major elements (S, K, Mg, P, Na, and Ca), samples of the culture medium were taken periodically and measured using inductively coupled plasma atomic emission spectroscopy in an ICAP—6000 Thermosystem (Thermo Electron Corporation, Waltham, MA, USA).

### 2.4. Media and Growth Conditions of Bacterial Strains

Stock cultures were grown in the Schlegel’s mineral medium [59,73] with an initial concentration of fructose or glucose of 5–10 g/L. The source of nitrogen was urea, whose concentration was 1 g/L in the stock culture inoculum and 0.5 g/L per 1 g ODW cell biomass in the PHA synthesis phase (concentration limiting cell growth) [73,74]. Fructose and/or glucose, which had been sterilized by membrane filtration (Opticap XL300 Millipore Express SHC filters, Merck, Darmstadt, Germany), served as carbon source in the initial medium. JA hydrolysates were used as sugar-containing substrates. Homopolymer of 3-hydroxybutyric acid was synthesized from a sole carbon source. Synthesis of PHA copolymers P(3HB-co-4HB) or P(3HB-co-3HV) was achieved as follows: after 18 h or 18 h and 40 h of cultivation, the culture medium was supplemented with precursor substrates (ε-caprolactone, and valeric acid in the form of potassium salt at a concentration of 1.0–2.0 g/L).

Cells were grown in batch culture, as developed previously for PHA synthesis [56]. A two-stage process was used. In the first stage, cells were grown under nitrogen deficiency: the amount of nitrogen supplied in this stage was 60 mg/g cell biomass synthesized (i.e., 50% of the cell’s physiological requirements—120 mg/g); the cells were cultured in complete mineral medium and with hydrolysate flux regulated in accordance with the requirements of the cells. In the second stage, cells were cultured in nitrogen-free medium; the other parameters were the same as in the first stage. The temperature of the culture medium was 30 °C and pH was 7.0 [73]. Inoculum was produced using an InnovaH 44 constant temperature incubator shaker (New Brunswick Scientific, Edison, NJ, USA). Inoculum was prepared by resuspending the stock culture maintained on agar medium. The stock culture was grown in 1–2 L glass flasks half-filled with mineral solution, with the initial concentration of glucose or fructose 10–15 g/L.

Cultivation was performed in a BioFlo-115 automated fermentation system (Eppendorf, Germany) with a 12-L fermentation vessel and the working volume of the culture from 4 to 8 L, under strictly aseptic conditions. The procedure of culturing bacteria in it was described elsewhere [56]. The fermenter was equipped with two turbine-type impellers: di = 0.075 m, the width of the impeller blade 0.030 m, the number of baffles in the fermenter 4, diameter and number of sparger holes 0.001 m and 10, respectively, jacket surface area 0.2 m^2^. The mass flow of the fermenter was controlled by the air flow rate and agitation speed; the latter could be varied from 300 to 1000 rpm, and, thus, the oxygen transfer rate, KLa, varied from 120 to 480 L/h. The fermenter was equipped with a control station with a liquid crystal display, which recorded the data of cultivation process, pH probes, O_2_ probes, a system for automatic substrate feeding, and a thermal stabilization system.

### 2.5. Monitoring Process Parameters

During the course of cultivation, samples of culture medium were taken for analysis every 4–5 h (from the culture in the fermenter); cell concentration in the culture medium was determined based on the weight of the cell samples dried at 105 °C for 24 h (CDW). Cell concentration in the culture medium was monitored every hour by converting the optical absorbance at 440 nm of culture broth to cell dry weight by using a standard curve prepared previously [73].

The cell dry weight (CDW) from fructose cultures was determined by centrifuging 20 mL of culture medium at 6000× *g* for 10 min and washing it twice with distilled water. After final centrifugation, the cells were re-suspended in 10 mL of distilled water, transferred into the preweighed weighing bottles, and dried at 105 °C for 24 h [73].

The parameters used to estimate culture growth and PHA synthesis were cell concentration in the culture medium and polymer content, consumption of the main growth substrate, and process duration and productivity [74]. Kinetic and production parameters of the culture were determined using conventional methods. The biomass concentration (X, kg/m^3^), the yields of the culture (Y_x_, g biomass/g substrate) and polymer (Y_p_, g PHA/g substrate), the specific growth rate (μ, h^−1^), and productivity (P, g/L per h) were calculated.

Specific growth rate of the culture (μ, h^−1^) was determined using the following equation:

μ = dX_c_ dt∗/X_c_ (1)

where X_c_ is catalytic biomass, g/L; t—duration of cultivation, h.

Specific rate of polymer synthesis (μβ, h^−1^) was determined using the following formula:

μβ = dPHA dt∗1/X_c_ (2)

where PHA denotes initial and final intracellular polymer concentrations, g/L.

Biomass and polymer fermentation productivity were determined using the formula

P_x_ = (X_n_ − X_0_)/T P_p_ = (PHA_n_ − PHA_0_)/T (3)

where X_0_, X_n_, PHA_0_, PHA_n_ are concentrations of biomass and polymer at the start and end of fermentation, respectively, g/L, T is fermentation duration h^−1^.

The yield of the polymer, Y_p_, was calculated using the following formula:

Y_p_ = ΔPHA/∆S (4)

where P is initial and final polymer content, g, and S is consumed substrate, g.

### 2.6. PHA Recovery from Cell Biomass

Polymer recovery was performed in two stages. First, lipids and fatty acids were removed using ethanol and, then, polymer was extracted with dichloromethane. The dichloromethane extracts were pooled and evaporated twice using an R/210V rotary evaporator (Büchi, Flawil, Switzerland). Then, polymer was precipitated with hexane, 1:2. Polymer content in the residual mass was determined using a 7890A gas chromatograph equipped with a 5975C chromatograph-mass spectrometer (Agilent Technologies, Santa Clara, CA, USA). Polymer was re-dissolved in chloroform several times and precipitated using isopropanol or hexane to purify it. The resulting polymer was dried at 40 °C [75].

### 2.7. PHA Chemical Composition

Intracellular content of the polymer and its composition were determined by chromatography of methyl esters of fatty acids after methanolysis of cell biomass using a 7890A chromatograph-mass spectrometer (Agilent Technologies, Santa Clara, CA, USA) equipped with a 5975C mass detector (Agilent Technologies, Santa Clara, CA, USA) [76]. Methanolysis of the samples was conducted as follows: 1 mL chloroform, 0.85 mL methanol, and 0.15 mL concentrated sulfuric acid were added to a 4.0–4.5-g polymer sample and boiled under reflux condensers for 160 min. At the end of methanolysis reaction, 1 mL distilled water was added to the flask. The bottom chloroform layer was used for analysis by chromatography [76].

### 2.8. Physicochemical Properties of PHAs

Physicochemical properties of PHAs were examined using high performance liquid chromatography, X-ray structure analysis, and differential scanning calorimetry and thermogravimetric analysis; methods and instruments have been described in detail elsewhere [73,74].

PHA properties were studied using high-purity polymers synthesized from different types of JA hydrolysates. Molecular-weight properties of PHAs were examined with a gel permeation chromatograph (Agilent Technologies 1260 Infinity, Waldbronn, Germany). Weight average molecular weight (M_w_), number average molecular weight (M_n_), and polydispersity (Ð) were measured.

Melting point (T_melt_) and thermal degradation temperature (T_degr_) were measured using a DSC-1 differential scanning calorimeter (Mettler Toledo, Schwerzenbac, Switzerland) and TGA (Mettler Toledo, Schwerzenbac, Switzerland), respectively. A 3–5 mg sample was heated to 200 °C at a rate of 5 °C/min; the sample was held at 200 °C for 1 min, cooled to −20°C at a rate of 5°C/min and held for 4 min. Then, the sample was reheated at a rate of 5 °C/min (DSC). A 3–5 mg sample was heated to 450 °C at a rate of 10 °C/min (TGA). Thermograms were analyzed using the “STARe v11.0” software.

X-ray structure analysis was performed to determine crystallinity of copolymers employing a D8 ADVANCE X-ray powder diffractometer equipped with a VANTEC fast linear detector (Bruker AXS, Karlsruhe, Germany). The degree of crystallinity (Cx) was calculated as a ratio of the total area of crystalline peaks to the total area of the radiogram (the crystalline + amorphous components). Calculations were done by using the Eva program of the diffractometer software.

### 2.9. Production and Investigation of Polymer Films

Films were prepared by casting a 2% polymer solution in dichloromethane in degreased Teflon-coated molds, and then the films were left to stay in a laminar flow cabinet (Labconco, Kansas City, MO, USA) for 72 h at room temperature.

The surface microstructure of PHA films was analyzed using scanning electron microscopy (FE-SEM S 5500 high-resolution scanning electron microscope, Hitachi, Tokyo, Japan). Prior to microscopy, the samples were sputter coated with platinum (at 25 mA, for 60 s), using an EM ACE200 (Leica, Vienna, Austria).

The roughness of film surface was determined using atomic-force microscopy (AFM) in semicontact mode (DPN 5000, NanoInk, Skokie, IL, USA). The arithmetic mean surface roughness (Sa) and the root mean square roughness (Sq) were determined based on 10 points, as the arithmetic averages of the absolute values of the vertical deviations of the five highest peaks and lowest valleys from the mean line of the surface profile, using conventional equations [77]. AFM data were processed, and statistical analysis of the images was performed using the Gwyddion (2.51) free software.

### 2.10. Statistics

ACM data were processed using free software Gwyddion. To obtain statistical values (Sa, Sq, Sz), each of the films was scanned in four different regions. After the statistical values were obtained using the Gwyddion instrument, the data were averaged. Porosity of the films was determined manually from SEM images using a software package for digital image analysis (free open-source software package for scientific analysis, editing, and processing of raster images), Image Jv1.52. Statistical analysis of the results was performed using the standard software package of Microsoft Excel. Arithmetic means and standard deviations were found.

## 3. Results and Discussion

### 3.1. Characterization of Hydrolysates of the JA tubers and Vegetative Biomass

Analysis of the JA chemical composition showed that tubers contained greater amounts of solids compared to vegetative parts and higher percentages of easy-to-hydrolyze polysaccharides and oligosaccharides (Table 1). Hard-to-hydrolyze polysaccharides, lignins, and oligosaccharides prevailed in leaves and stems.

JA tubers are rich in sugars (Table 1). They are mainly represented by easy-to-hydrolyze polysaccharides, which constitute 34.1%—a considerably higher percentage compared to the vegetative part. Hard-to-hydrolyze polysaccharides amount to just 12.3%. In the vegetative part, hard-to-hydrolyze polysaccharides constitute 23.5%, and the portion of easy-to-hydrolyze ones is no more than 10%. Thus, sugars constitute more than 60% of the JA tubers and 50% of the JA vegetative part, making this crop an attractive inexpensive carbohydrate source for biotechnology.

The composition of JA hydrolysates is presented in Table 2.

The RS content of the JA tuber hydrolysates ranged between 50.3 and 73.2% depending on the hydrolysis procedure, and the RS were mainly represented by monosaccharides. The simultaneous extraction and acid hydrolysis at 80 °C resulted in higher RS contents. The major monosaccharide of tuber hydrolysates was fructose. Glucose was present in insignificant quantities. The second procedure of hydrolysis resulted in higher amounts of glucose, suggesting partial hydrolysis of the hard-to-hydrolyze polysaccharides (fiber and cellulose). Hydrolysates of the JA vegetative biomass contained considerably smaller amounts of RS, but the monosaccharides contained almost equal percentages of glucose and fructose as well as galactose.

The hydrolysates produced by aqueous extraction followed by sugar hydrolysis at 60 °C contained all micro- and macronutrients necessary for the growth of microorganisms (Table 3). However, the acid hydrolysates produced at 80 °C contained increased levels of Cr, S, Ni, Fe, Mn, and Ca, which could inhibit cell growth. Moreover, acid hydrolysis at elevated temperatures induces secondary oxidation of fructose and formation of furfural, formaldehyde, phenol, and other toxic compounds unfavorable for microorganisms [64,65,66,67]. Therefore, before the acid hydrolysates can be safely used, they will likely require further treatment to remove excess mineral compounds and toxic organic compounds.

Conditions and parameters of hydrolysis of plant material largely determine the composition of the resulting hydrolysates [36,39,68,69,78]. The hydrolysates of the JA tubers and vegetative biomass produced using the hydrolysis procedures described above contained RS and sugars, including fructose and glucose, at concentrations similar to those reported by other authors (Table 2 and Table 3) [52,53]. According to the available literature data on fermentative hydrolysis of JA, the highest fermentative conversions of inulin to fructose and glucose (more than 70%) were achieved in solid state fermentation of the *A. awamori* fungus using ground JA tubers as substrate aiming at production of invertase and inulinase [36]. That result was attained by revealing optimal parameters of hydrolysis (process duration, degree of grinding, and moisture content of the biomaterial). The highest degree of inulin hydrolysis and concentrations of fructose (32.6 ± 2.3 g/L) and glucose (4.7 ± 0.1 g/L) in hydrolysate were achieved after 48 h; then, hexose concentrations decreased. Another factor influencing conversion of inulin to sugars is the type of the microorganism employed and the activity of the enzymes in it. The efficiency of inulin hydrolysis at 50 °C varied between 34.7 and 87.9% when different inulinase sources were used (*A. niger* A42, *K. marxianus* NCYC 587) [79] or when mixed inulinases from *Aspergillus niger* and *Candida guilliermondii* were employed [80].

### 3.2. Production Parameters of PHA Synthesis from Jerusalem Artichoke Hydrolysates

Preliminary results of cultivation of three closely related bacterial strains on JA hydrolysates in flasks are presented in Table 4. All types of sugar-containing substrates produced using different hydrolysis procedures from both JA tubers, which were mainly represented by fructose, and JA vegetative biomass, which contained less RS but almost equal amounts of glucose and fructose, were suitable for growing the strains used in this study (*C. necator* B-10646 and *R. eutropha* B5786 and B8562). At the same time, cell growth and polymer synthesis differed across strains: total biomass concentration varied by a factor of almost two, from 3.4 to 7.0 g/L, and polymer content from 49.1 to 70.0%. The process was the most efficient in *C. necator* B-10646 culture while *R. eutropha* B 5786, which metabolizes only fructose for growth, showed the poorest results. The differences obtained for the study strains were in good agreement with the data reported in the study of chicory root hydrolysates used to synthesize PHAs [51]. The authors of that work found considerable differences between total biomass concentrations and polymer contents of three strains of one bacterial species grown on the same hydrolysate type (Table 4).

Parameters were reduced when cells were cultivated on acid hydrolysates produced at 80 °C while whether hydrolysates had been produced from JA tubers or stems and leaves was not a major factor. All strains produced considerably lower biomass concentrations, but polymer content was lower to a lesser extent, probably because sugar-containing substrates produced by acid hydrolysis at 80 °C contained excess minerals and organic compounds inhibiting cell growth such as phenol, furfural, etc.

Parameters of cell growth and PHA synthesis obtained for three strains (*C. necator* B-10646 and *R. eutropha* B5786 and B8562) cultivated on JA hydrolysates were comparable to our preliminary results [53], which demonstrated for the first time that *Ralstonia eulropha* Z-l was able to synthesize P(3HB) amounting to 60–70% when cultivated on hydrolysates of JA vegetative biomass (Table 4). Comparison of the results obtained on JA hydrolysates with our data obtained on pure sugars showed that biomass concentrations produced by the three study strains, especially *C. necator* B-10646, were comparable to each other. Polymer contents were, however, lower than the polymer content produced by the *C. necator* IBP/SFU-1 whose biomass concentrations on fructose and glucose were 7.0 and 7.7 g/L, respectively [82]. Results obtained on JA hydrolysates were also somewhat lower than production parameters of *C. necator* B-10646 on commercial pure sugars (glucose and fructose): over 72-h cultivation, biomass concentration reached 8.4 g/L and PHA content 92% [83]. Nevertheless, results obtained in the present study were similar to or higher than the data reported in other works for such strains as *Alcaligenes eutrophus* H16 [84,85], *R. eutropha* NCIMB 11599 [86], *R. eutropha* B8562 [56], and *Cupriavidus necator* A-04 [87]. In previous research of the strains used in the present study, cultural properties of the glucose-utilizing mutant strain *Ralstonia eutropha* B8562 were compared with those of its parent strain *R. eutropha* B5786. It was shown that growth characteristics of the strain cultured on glucose as the sole carbon and energy source were comparable with those of the parent strain. The strain is regarded as a promising PHA producer on available glucose-containing media [56].

**Figure 2:**
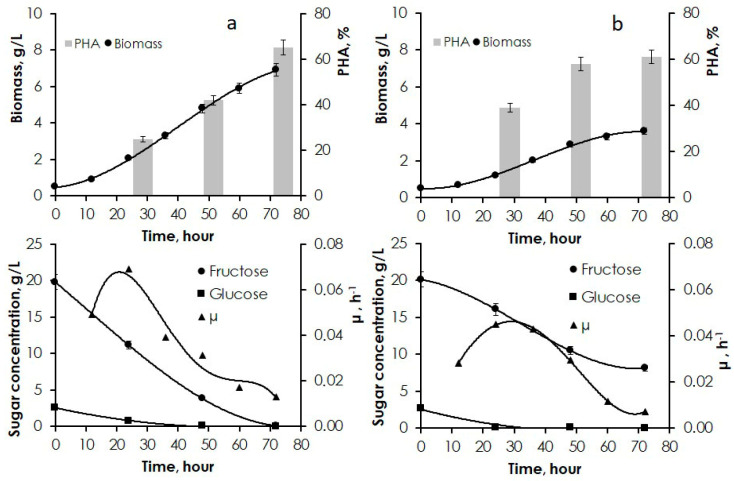
Parameters of cell growth and PHA synthesis achieved by *C. necator* B-10646 cultivated on JA tuber hydrolysates (growth curve, PHA content, consumption of carbohydrate substrates, specific growth rate): (**a**) aqueous extraction at 80 °C followed by acid hydrolysis at 60 °C, (**b**) simultaneous extraction and acid hydrolysis at 80 °C.

**Figure 3:**
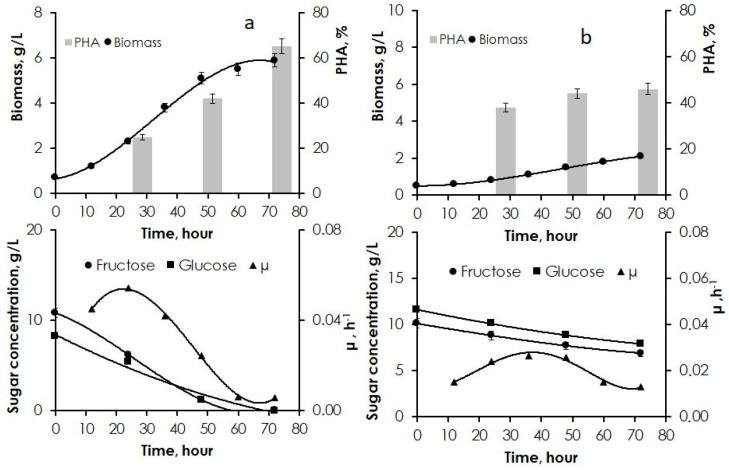
Parameters of cell growth and PHA synthesis achieved by *C. necator* B-10646 cultivated on JA vegetative biomass hydrolysates (growth curve, PHA content, consumption of carbohydrate substrates, specific growth rate): (**a**) aqueous extraction at 80 °C followed by acid hydrolysis at 60 °C, (**b**) simultaneous extraction and acid hydrolysis at 80 °C.

Thus, preliminary research showed that JA tuber and vegetative biomass hydrolysates were suitable substrates for PHA synthesis. Then, the most productive strain, *C. necator* B-10646, was cultivated in larger (2-L) flasks to investigate cell growth and PHA synthesis parameters in more detail (Figure 2 and Figure 3).

The best results were obtained in fermentations on JA tuber hydrolysate prepared by the first hydrolysis procedure, i.e., by aqueous extraction of sugars at 80 °C followed by acid hydrolysis at 60 °C. Total biomass concentration reached 6.9 g/L within 72 h and polymer content was 69%—results comparable to those obtained in fermentations on pure sugars. Specific growth rate was 0.07 h^−1^ (Equation (1)), with the highest value achieved in the first phase of the process (at 22–25 h) (Figure 2a). Sugars were consumed by the cells evenly. Glucose, whose concentration was one order of magnitude lower than fructose concentration in tuber hydrolysates, was completely utilized by cells after 48 h of cultivation. Residual fructose concentration by the end of cultivation was very low (0.1 g/L). The yield from the consumed sugars was 0.3 g biomass/g sugar.

Cultivation on the acid hydrolysate of JA tubers produced at 80 °C resulted in a decrease in biomass concentration to 3.6 g/L and specific growth rate in the exponential phase to 0.04 h^−1^ (Equation (1)); the final polymer content was 61% (Figure 2b). Fructose consumption was reduced as cell growth slowed down. Residual glucose concentration was 8.1 g/L. Glucose, whose concentration in hydrolysate was 2.7 g/L, was completely utilized within the first hours of the process.

Similarly to what was found for tuber hydrolysates, bacterial cultivation on hydrolysates of the JA vegetative biomass produced by aqueous extraction of sugars at 80 °C followed by weak-acid hydrolysis at 60 °C yielded better results (Figure 3a). Biomass concentration was 5.9 g/L, and intracellular PHA content was 65%. The growing cells evenly consumed glucose and fructose. The yield from glucose and fructose was 0.31 g biomass/g sugar.

Results of using hydrolysates of JA vegetative biomass containing similar concentrations of glucose and fructose are shown in Figure 3b. Cultivation of *C. necator* B-10646 on the sugar-containing substrate produced by acid hydrolysis of the vegetative part resulted in the lowest biomass concentration (up to 2 g/L) and polymer content (below 50%). The inhibition of the growth of bacterial cells cultivated on acid hydrolysates of the JA vegetative part (similar to the acid hydrolysates of JA tubers) was caused not only by the excess of some minerals but also, apparently, by processes of secondary conversion of monosaccharides at 80 °C, which led to accumulation of contaminants (furfural, hydroxymethylfurfural, methylfurfural, formaldehyde, phenols) in the culture medium [67]. The lower contents of PHAs synthesized from sugar-containing JA products compared to the results of the synthesis on pure sugars could also be attributed to the presence of reduced nitrogen, NH_4_^+^, in the extracts, which amounted to 0.74–0.89 g/L in the aqueous extracts prepared from JA tubers and 0.45–0.52 g/L in the extracts from the JA vegetative part.

Both results of the present study and literature data demonstrated that cell growth on pure sugars yielded somewhat higher production parameters than cultivation on hydrolysates. A study by Koutinas et al. (2013) found that JA hydrolysates supplemented with the yeast extract considerably influenced P(3HB) contents, which varied between 15.2 and 51.9%. Moreover, the biomass concentration was lower compared to cultivation on commercial fructose [36] (Table 4). The authors compared the results of that study with their previous studies and concluded that production parameters of cultivation on JA hydrolysates were comparable with the P(3HB) production by *C. necator* NCIMB 11599 from wheat hydrolysates and *C. necator* DSM 545 from crude glycerol mixed with rapeseed cake hydrolysates [81], but were somewhat lower than the parameters obtained on the media with glucose and glycerol.

In another study [39], polymers were synthesized by *C. necator* DSM 428 on the medium containing inulin and a fungal inulinase mixture produced in Penicillium lanosocoeruleum culture. The authors noted high efficiency of inulin conversion (about 93%) into a mixture of sugars (30 g/L) that mainly consisted of fructose and a minor fraction of glucose (3 g/L), which is not utilized by the study strain. Cell biomass and polymer concentrations at 96 h of cultivation were 3.9 g/L and 82%, respectively (Table 4). The production parameters of that process were lower than the values reported by other authors and results obtained in the present study.

**Figure 4:**
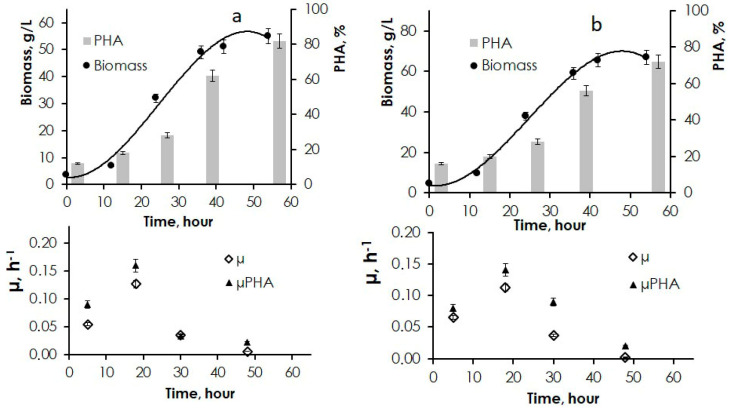
Production parameters of *C. necator* B-10646 cultivated on hydrolysates of (**a**)—JA vegetative biomass and (**b**)—JA tubers (aqueous extraction at 80 °C followed by acid hydrolysis at 60 °C): biomass concentration (X, g/L) and intracellular polymer content (PHA% of ODW); specific growth rate of cells (µX h^−1^) and specific rate of polymer synthesis (µPHA, h^−1^).

As the best results were obtained with the JA hydrolysates produced by aqueous extraction (80 °C) followed by acid hydrolysis (60 °C), these substrates were used to scale up the process to cultivation of bacterial cells in fermentation systems. Figure 4a shows results of cultivation of *C. necator* B-10646 on hydrolysates of the JA vegetative part as the carbon substrate.

With the initial cell concentration in the inoculum 2.5 g/L, total biomass concentration reached 55.1 g/L within 55 h. After 36 h of the experiment, polymer content was 62%; the specific growth rate was the highest after 18 h, reaching 0.13 h^−1^ (Equation (1)), but after 36 h, as intracellular polymer concentration increased, specific growth rate dropped to 0.03 h^−1^. By the end of the experiment, intracellular polymer content had reached 76%. The highest specific rate of PHA synthesis was recorded after 18–20 h (0.16 h^−1^) (Equation (2)); by the end of the experiment, specific rate of PHA synthesis had dropped to 0.02 h^−1^. Specific rate of sugar (fructose and glucose) consumption reached its maximum in the exponential phase, between 12 and 30 h, amounting to 3.1 to 3.3 g/g·h; then, sugar consumption by the cells decreased, and after 60 h, specific rate of sugar consumption was 0.012 g/g·h. The polymer yield from sugars and process productivity were 0.24 g/g (Equation (4)) and 0.76 g/L·h (Equation (3)), respectively.

Similar biomass concentrations but higher polymer contents were achieved in the experiment with JA tuber hydrolysates used as carbon substrate to grow *C. necator* B-10646 (Figure 4b). Within 55 h of cultivation, biomass concentration reached 66.9 g/L. After 36 h, polymer content was 56%, increasing to 82% by the end of the experiment. The highest specific growth rate of the cells was recorded in the exponential phase (12–30 h): 0.11 h^−1^ (Equation (1)). After 30 h, as the polymer content increased, specific growth rate decreased to 0.03 h^−1^, and at the end of the experiment, it was only 0.002 h^−1^. The highest fructose consumption rate was observed in the first phase of the process, with the maximum in the exponential phase of cell growth (3.2–3.4 g/g·h). In the second phase, in the nitrogen-free culture medium, when intracellular PHA content was growing, fructose consumption decreased, dropping to 0.008 g/g·h by the end of the experiment. The polymer yield from fructose and productivity of *C. necator* B-10646 culture were 0.25 g/g (Equation (4)) and 1.00 g/L·h (Equation (3)), respectively; that is, polymer production by the strain from this carbon source was higher.

Unfortunately, there are no available studies describing bacterial cultivation on JA and/or chicory root hydrolysates in rather large fermentation systems, and, thus, we cannot compare our results with the data of other researchers. Results obtained in the present study are comparable to our previous study of *C. necator* B-10646 cultivated under similar conditions in a 12-L Bio-Flo fermenter. With the same initial biomass concentration in the inoculum (2.5 g/L), cells were grown in the glucose-containing medium under limited nitrogen supply in the first phase and in the nitrogen-free medium in the second; biomass concentration reached 62.0 g/L. The process productivity was 1.24 g/L·h of cells and 0.99 g/L·h of PHA, respectively. Glucose consumption averaged 2.56 ± 0.1 g/g cells and 3.56 ± 0.1 g/g polymer [88].

Thus, the integrated research carried out in this study showed that sugar-containing hydrolysates of JA tubers and vegetative biomass can be used as carbon substrates enabling productive biosynthesis of PHAs.

### 3.3. Structure and Properties of PHAs Synthesized from Jerusalem Artichoke Hydrolysates

Properties of PHAs are determined by their structure, primarily, the structure of the side chains in the polymer carbon chain and the distance between the ester groups in a molecule. Carbon source is one of the cultivation conditions considerably influencing the chemical composition and properties of PHAs.

The few studies [36,39,51] addressing PHA synthesis on JA hydrolysates do not provide any data on polymer properties. In the present study, the effects of JA hydrolysates on physicochemical properties of the polymers were investigated for the first time. Polymer specimens examined in this study were synthesized in the most productive processes, from hydrolysates of JA tubers and vegetative biomass produced by aqueous extraction at 80 °C followed by acid hydrolysis at 60 °C (Table 5).

**Figure 5:**
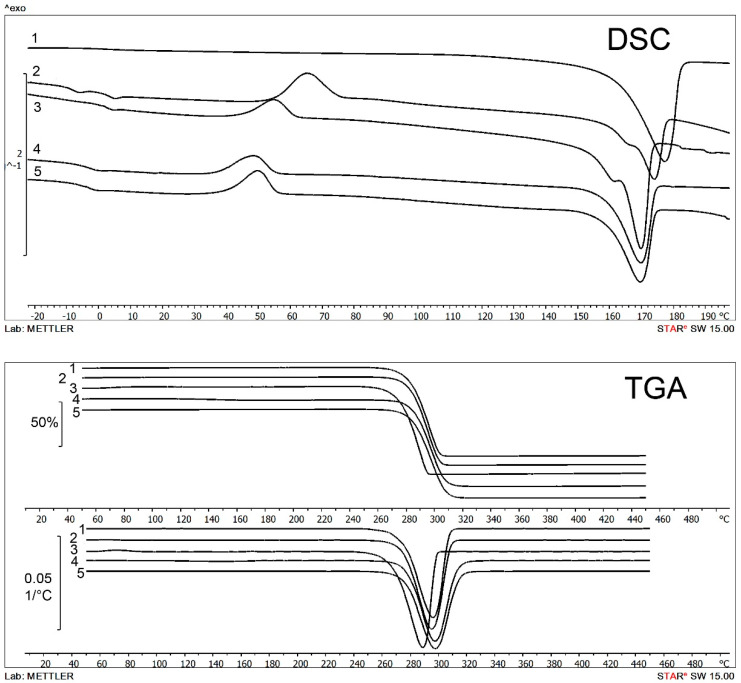
Results of thermal analysis of PHAs synthesized by *Cupriavidus necator* B-10646 from hydrolysates of JA tubers. 1-P(3HB), 2-P(3HB-co-37.4 mol.%3HV), 3-P(3HB-co-9.9 mol.%3HV), 4-P(3HB-co-11.9 mol.%4HB), 5-P(3HB-co-21.1 mol.%4HB).

The properties of all P(3HB) specimens examined in this study corresponded to the generally accepted properties of this PHA, regardless of the source (tubers or vegetative biomass), hydrolysis conditions, and PHA producing strain employed. All specimens exhibited the 100–120 °C difference between the melting point and degradation temperature (Table 5, Figure 5); prevailing of the crystalline phase over the amorphous one and degrees of crystallinity between 69 and 75%; and variations in weight average molecular weight—409–480 kDa.

Variations in the values of molecular weight of the P(3HB) specimens are consistent with the existing data on considerable variations in these parameters, which are associated not only with the specific culture medium and differences between bacterial strains but also with the duration of fermentation, type of reagents, recovery techniques, degree of purity of the specimens, etc.

One of the most valuable properties of PHAs is their diversity: they can vary in their chemical composition and basic characteristics (degree of crystallinity, molecular weight and thermal properties, degradation behavior in biological media). The strain *C. necator* B-10646 is tolerant to precursor substrates of different monomers and capable of synthesizing copolymers composed of various monomer units. For example, cultivation of this strain in the medium with glucose as the main carbon source supplemented with precursor substrates (valerate, hexanoate, propionate, γ-butyrolactone) enabled synthesis of PHA bi-, ter-, and quaterpolymers [88,89].

Supplementation of the culture medium of *C. necator* B-10646 grown on JA hydrolysates with potassium valerate and ε-caprolactone resulted in the synthesis of P(3HB-co-3HV) and P(3HB-co-4HB) copolymers with 3HV and 4HB percentages depending on how many times the precursors were added to the medium (Table 5). The percentage of the monomers other than 3HB affected, first of all, the degree of crystallinity of the polymers, which decreased as the fraction of the second monomer increased. That was more noticeable in P(3HB-co-4HB) specimens, whose Cx decreased to 54 and 46% at 4HB 11.9 mol.% and 21.1 mol.%, respectively. The P(3HB-co-3HV) copolymers exhibited a similar, although less obvious, trend. The degree of crystallinity of P(3HB-co-3HV) containing 3HV 9.9 and 37.4 mol.% was 60 and 49%, respectively. All copolymers showed an insignificant increase in Mw and Mn. Copolymers containing higher percentages of 3HV and 4HB had lower melting points and thermal degradation temperatures. The differences in the properties between PHA copolymers and P(3HB) were characteristic of PHAs and consistent with the data reported in other studies [8,10,11,14,16,88,89].

**Figure 6:**
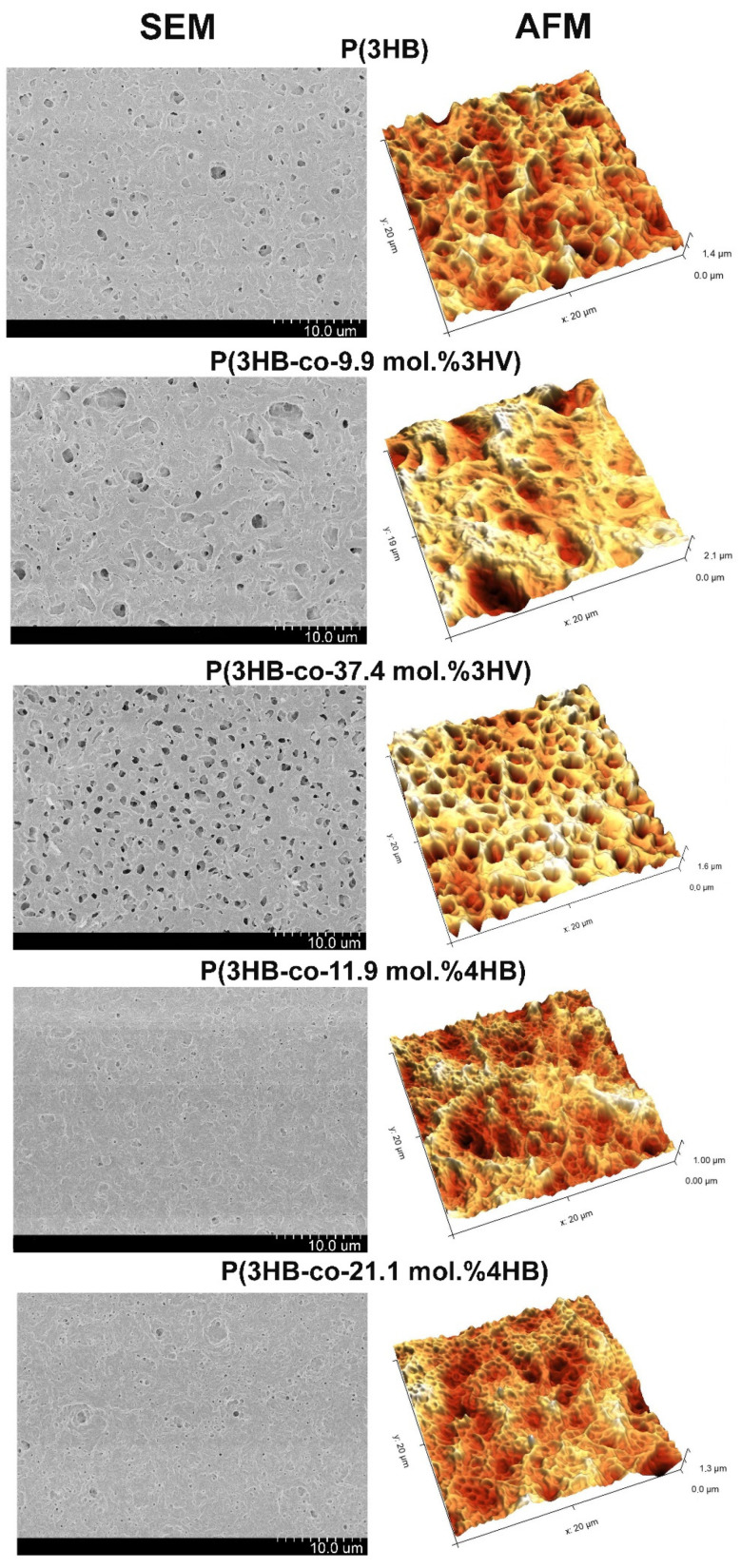
SEM and AFM images of PHA specimens synthesized from JA hydrolysates.

The differences in the physicochemical properties of PHA specimens synthesized from JA hydrolysates influenced the properties of the polymer films produced by solution casting technique (Figure 6 and Table 6). The most pronounced differences were observed in film porosity and surface roughness.

Nanometer roughness determines protein adhesion, cell attachment, growth, and synthesis of specific proteins. Examination of polymer films of using atomic force microscopy showed the effect of the chemical composition of PHAs on surface roughness (Figure 6 and Table 6). The values of arithmetic mean surface roughness (Sa), root mean square roughness (Sq), and peak-to-valley height (Sz) for P(3HB) film were 163, 211, and 1047 nm, respectively (Table 6). The obtained values were close to those for the film of P(3HB) synthesized by the strain *C. necator* B-10646 from glucose [89]. Arithmetic mean surface roughness (Sa) is similar to root mean square roughness (Sq), and the difference is that it is calculated as total difference modules between the data value and the mean rather than squared difference. Peak-to-valley height (Sz) comprises the full range of values; this is total difference between the profile valleys and peaks (between the lowest valley (Sv) and Sp (the highest peak). Films made from copolymers with 3HV had higher values of these parameters, which indicated greater roughness of these films. By contrast, for films with 4HB, the values of Sa, Sq, and Sz were lower: 112–131, 142–168, and 605–641 nm, respectively. Results of this study are in good agreement with the data reported in other works, which also show that incorporation of 3HV results in production of films with a rougher surface [89,90,91,92] compared to the films made of P(3HB). However, no direct relationship was found between Sa, Sq, and Sz and the fraction of 3HV. The studies of copolymers with 4HB yielded contradictory results. In the current study, roughness parameters of the films made from copolymers with 4HB were lower compared to the films of P(3HB), and that was consistent with the data reported in a study by Chanprateep et al. (2010) [93]. There are studies, however, demonstrating that P(3HB-co-4HB) films had rougher surface [92]. It is well-known that even small variations in the film surface profile may cause different cellular response: from an insignificant increase to a considerable inhibition of cellular activity.

Thus, the use of PHAs of various chemical composition makes it possible to fabricate films with various degrees of porosity and surface development.

Analysis of porosity demonstrated that the surfaces of the PHA films differed significantly in the pore number and average pore area (Table 6). That was most likely associated with differences in the kinetics of crystallization during the formation of films as they dried and the solvent evaporated. The number of pores in P(3HB) films was 20.8 pores/1000 µm^2^ with an average pore area of 7.9 µm^2^. The surface of all copolymer films, except the film fabricated from P(3HB-co-9.9 mol.%3HV), had pores with a smaller area, from 1.7 to 4.7 µm^2^. Films of P(3HB-co-9.9 mol.%3HV) had larger pores (with an average area of 30.8 µm^2^), but their number was comparable to the number of pores in the P(3HB) film. Films with higher contents of 3HV (37.4 mol.%) and 4HB (21.1 mol.%) contained a larger number of pores: 49.6 and 103.8 pores/1000 µm^2^, respectively. These differences were reflected in the integrated indicator—the total pore area. The lowest values of the total pore area, 77 µm^2^/1000 µm^2^ of the film surface area, were found for films fabricated from P(3HB-co-11.9 mol.%4HB) (Table 6). The total pore areas found for P(3HB), P(3HB-co-21.1 mol.%4HB), and P (3HB-co-37.4 mol.%3HV) films were 164, 173, and 235 μm^2^/1000 μm^2^, respectively. The pore area of the P(3HB-co-9.9 mol.%3HV) film was several times larger—788 μm^2^/1000 μm^2^.

The effect of PHA composition on film porosity was also shown in other studies [89,92]. In addition, the study by Zhila et al. (2021) demonstrated that porosity of the films, namely, the total pore area, which indicates the number of pores and their area, differed several-fold between the films of P(3HB) synthesized from different substrates (CO_2_, glucose, fructose, saturated fatty acids C12-C18) [82]. Thus, results of the present study are in good agreement with the data suggesting the effect of PHA composition on the surface structure of polymer films.

The study showed that hydrolysates of JA tubers and vegetative biomass used as carbon source enabled productive synthesis of PHAs, comparable to PHA synthesis from pure sugars. The next step is to scale up PHA synthesis from this effective substrate and conduct the feasibility study.

## 4. Conclusions

The present study investigated bacterial cell growth and PHA accumulation on JA hydrolysates. Comparison of two procedures of tuber and vegetative biomass hydrolysis that differed in the process temperature and duration showed variations in the RS contents (between 59.6–73.2 and 11.5–31%) and fructose to glucose ratios of the hydrolysates, depending on the hydrolysis procedure employed. All types of hydrolysates were tested as substrates for the strains *Cupriavidus necator* B-10646 and *Ralstonia eutropha* B5786 and B8562 and found to be suitable for cell growth and PHA synthesis, although fermentations resulted in different polymer contents and, in particular, different biomass concentrations. The most effective PHA synthesis, conducted in 12-L fermenters, was achieved on hydrolysates of JA tubers (X = 66.9 g/L, 82% PHA) and vegetative biomass (55.1 g/L and 62%) produced by aqueous extraction of sugars at 80 °C followed by weak-acid hydrolysis at 60 °C. Biomass concentrations produced in experiments with hydrolysates prepared at 80 °C, especially vegetative biomass hydrolysates, were considerably lower, although the difference in polymer contents was somewhat smaller. The most likely reason was that cell growth was inhibited by excess concentrations of minerals and toxic compounds such as phenols and furfural—products of fructose hydrolysis at high temperatures. Kinetic and production parameters of the process were studied for the four types of JA hydrolysates using the most productive strain, *C. necator* B-10646. The effects of JA hydrolysates on physicochemical properties of the polymers were studied for the first time. The properties of all P(3HB) specimens synthesized from JA hydrolysates corresponded to the generally accepted parameters of this PHA, regardless of the source (tubers or vegetative biomass), hydrolysis conditions, and PHA producing strain employed. All specimens exhibited the 100–120 °C difference between the melting point and degradation temperature; prevailing of the crystalline phase over the amorphous one and degrees of crystallinity between 69 and 75%; and variations in weight average molecular weight—409–480 kDa. Supplementation of the culture medium of *C. necator* B-10646 grown on JA hydrolysates with potassium valerate and ε-caprolactone resulted in the synthesis of P(3HB-co-3HV) and P(3HB-co-4HB) copolymers with 3HV and 4HB percentages depending on how many times the precursors were added to the medium. PHA copolymers had reduced degrees of crystallinity and molecular weights, which influenced the porosity and surface roughness of polymer films prepared from them.

Thus, the process conducted in a 12-L fermenter on JA hydrolysates enabled productive synthesis of PHAs, comparable to PHA synthesis from pure sugars, suggesting good potential of this substrate for PHA production. The present study showed successful use of Jerusalem artichoke hydrolysates as substrate for productive synthesis of PHAs, contributing to the solution of the critical problem of PHA biotechnology—finding widely available and inexpensive substrates.

## Figures and Tables

**Table 1:** Chemical composition of Jerusalem artichoke cv. Interes.

Parameter	Content, %	
	Tubers	Vegetative Biomass
Moisture content	74.0 ± 3.7	12.0 ± 0.6
Solids including	26.0 ± 0.9	88.0 ± 4.4
Minerals	4.2 ± 0.3	8.5 ± 0.6
Monosaccharides	6.4 ± 0.6	7.9 ± 0.7
Oligosaccharides	12.7 ± 1.0	18.7 ± 1.1
Crude protein	2.5 ± 0.3	-
Pectic substances	1.2 ± 0.1	-
Easy-to-hydrolyze polysaccharides, % CDW	34.1 ± 2.4	10.7 ± 0.7
Hard-to-hydrolyze polysaccharides, % CDW	12.3 ± 0.9	23.5 ± 1.9
Lignins, % CDW	9.1 ± 0.4	16.2 ± 0.9

«-»—not determined.

**Table 2:** Composition of hydrolysates of the tubers and vegetative biomass of Jerusalem artichoke cv. Interes under different procedures of hydrolysis.

Parameter, %	Aqueous Extraction at 80 °C Followed by Acid Hydrolysis at 60 °C	Simultaneous Extraction and Acid Hydrolysis at 80 °C
Tuber hydrolysates:		
Reducing substances (RS)	59.8 ± 4.2	73.2 ± 5.1
Monosaccharides (% of RS):	49.1 ± 2.9	66.0 ± 3.3
galactose	-	-
glucose	4.3 ± 0.3	7.8 ± 0.4
fructose	44.7 ± 2.2	58.2 ± 2.3
Vegetative biomass hydrolysates:		
Reducing substances (RS)	31.0 ± 1.2	11.5 ± 0.7
Monosaccharides (% of RS):		
galactose	15.9 ± 0.8	1.1 ± 0.1
glucose	34.1 ± 1.3	44.6 ± 1.3
fructose	45.1 ± 1.2	38.1 ± 1.3

“-”—not detected.

**Table 3:** Mineral elements in hydrolysates of Jerusalem artichoke cv. Interes (mg/L).

Element	Tubers		Vegetative Biomass	
	Aqueous Extraction at 80 °C Followed by Acid Hydrolysis at 60 °C	Simultaneous Extraction and Acid Hydrolysis at 80 °C	Aqueous Extraction at 80 °C Followed by Acid Hydrolysis at 60 °C	Simultaneous Extraction and Acid Hydrolysis at 80 °C
S	59	1127	89	1324.00
P	286	211	121	110
K	2944	2900	1570	830
Na	32	35	30	79
Ca	73	88	122	121
Mg	72	112	135	138
Fe	0.07	4.9	5.8	48.0
Cu	0.23	0.38	0.27	0.46
Zn	0.05	2.1	0.75	5.11
Mn	0.17	1.12	1.55	14.3
Cr	0.0063	0.081	0.38	7.5
Ni	0.05	0.1	0.07	72.3
B	0.3	0.31	0.21	0.48
Mo	0.01	0.04	0.01	0.23

**Table 4:** Culture conditions, biomass concentrations, and PHA contents produced by different bacterial strains cultivated on hydrolysates.

PHA Producing Strain	X, g/L	PHA, %	Culture Conditions	Type of Hydrolysate	Reference
*Cupriavidus necator* B-10646	7.0	70.0	Shake flasks, 72 h	JA tuber hydrolysates, aqueous extraction at 80 °C followed by acid hydrolysis at 60 °C	This study
*Ralstonia eutropha* B5786	5.7	62.2	Shake flasks, 72 h	JA tuber hydrolysates, Aqueous extraction at 80 °C followed by acid hydrolysis at 60 °C	This study
*Ralstonia eutropha* B8562	6.9	67.8	Shake flasks, 72 h	JA tuber hydrolysates, Aqueous extraction at 80 °C followed by acid hydrolysis at 60 °C	This study
*Cupriavidus necator* B-10646	5.6	63.0	Shake flasks, 72 h	JA tuber hydrolysates, Simultaneous extraction and acid hydrolysis at 80 °C	This study
*Ralstonia eutropha* B5786	3.4	49.1	Shake flasks, 72 h	JA tuber hydrolysates, Simultaneous extraction and acid hydrolysis at 80 °C	This study
*Ralstonia eutropha* B8562	5.2	61.8	Shake flasks, 72 h	JA tuber hydrolysates, Simultaneous extraction and acid hydrolysis at 80 °C	This study
*Cupriavidus necator* B-10646	6.9	67.9	Shake flasks, 72 h	JA vegetative biomass hydrolysates, Aqueous extraction at 80 °C followed by acid hydrolysis at 60 °C	This study
*Ralstonia eutropha* B5786	5.3	57.7	Shake flasks, 72 h	JA vegetative biomass hydrolysates, Aqueous extraction at 80 °C followed by acid hydrolysis at 60 °C	This study
*Ralstonia eutropha* B8562	6.4	60.0	Shake flasks, 72 h	JA vegetative biomass hydrolysates, Aqueous extraction at 80 °C followed by acid hydrolysis at 60 °C	This study
*Cupriavidus necator* B-10646	5.6	64.3	Shake flasks, 72 h	JA vegetative biomass hydrolysates, Simultaneous extraction and acid hydrolysis at 80 °C	This study
*Ralstonia eutropha* B5786	3.4	48.9	Shake flasks, 72 h	JA vegetative biomass hydrolysates, Simultaneous extraction and acid hydrolysis at 80 °C	This study
*Ralstonia eutropha* B8562	5.3	60.8	Shake flasks, 72 h	JA vegetative biomass hydrolysates, Simultaneous extraction and acid hydrolysis at 80 °C	This study
*Ralstonia eutropha* Z-1	6–7	60–70	Shake flasks, 72–96 h	JA vegetative biomass acid hydrolysate	[53]
*Cupriavidus necator* DSM 428	11.3	66	Bioreactor, 120 h	Hydrolysate from chicory roots	[51]
*Cupriavidus necator* DSM 545	14	78	Bioreactor, 72 h	Hydrolysate from chicory roots	[51]
*Cupriavidus necator* DSM 531	3.5	46	Bioreactor, 120 h	Hydrolysate from chicory roots	[51]
*Cupriavidus necator* NCIMB 11599	3.5–20.8	10–70%	Shake flasks, 18–35 h	Wheat hydrolysates and fungal extract	[81]
*Cupriavidus necator* DSM 4058	5.3–9.2	15–52	Shake flasks, 32–56 h	JA tubers hydrolysate and yeast extract	[36]
*Cupriavidus necator* DSM 428	3.9	82	120 h	Inulin and fungal inulinase mixture	[39]

**Table 5:** Properties of P(3HB) homopolymer and PHA copolymers synthesized by *C. necator* B-10646, *R. eutropha* B5786 and B8562 from JA hydrolysates.

Strain	Culture Conditions	Number Average Molecular Weight, M_n_, kDa	Weigh Average Molecular Weight, M_w_, kDa	Polydispersity, Ð	Degree of Crystallinity, C_x_, %	Melting Point, T_melt_, °C	Thermal Degradation Temperature, T_degr_, °C
P(3HB)							
Hydrolysate of JA tubers							
*C. necator* B-10646	Shake flasks, 72 h	104	432	4.2	71	176	293
*R. eutropha* B5786	Shake flasks, 72 h	121	480	4.0	72	178	290
*R. eutropha* B8562	Shake flasks, 72 h	111	466	4.2	72	178	296
Hydrolysate of JA vegetative biomass							
*C. necator* B-10646	Shake flasks, 72 h	116	448	3.9	70	175	280
*R. eutropha* B5786	Shake flasks, 72 h	110	430	4.0	75	171	290
*R. eutropha* B8562	Shake flasks, 72 h	103	409	4.0	69	177	281
Hydrolysate of JA tubers							
P(3HB-co-9.9 mol.%3HV)							
*C. necator* B-10646	Shake flasks, 72 h; addition of sodium valerate (1 g/L)	129	620	4.8	60	170	275
P(3HB-co-37.4 mol.%3HV)							
*C. necator* B-10646	Shake flasks, 72 h; addition of sodium valerate (2 g/L)	194	1170	6.0	49	174	283
P(3HB-co-11.9 mol.%4HB)							
*C. necator B-10646*	Shake flasks, 72 h; addition of ε-caprolactone (2 g/L)	147	527	3.6	54	170	285
P(3HB-co-21.1 mol.%4HB)							
*C. necator B-10646*	Shake flasks, 72 h; addition of ε-caprolactone (4 g/L)	162	550	3.4	46	169	284

**Table 6:** Characterization of the films of PHAs synthesized from JA.

Porosity			Surface Roughness:		
Average Pore Area, µm^2^	Number of Pores, Pores/1000 µm^2^	Total Pores Area, µm^2^/1000 µm^2^	Arithmetic Mean Surface Roughness, (S_a_) nm	Root Mean Square Roughness, (S_q_) nm	Peak-to-Valley Height, (S_z_) nm
P(3HB)					
7.9	20.8	164	163.10	211.01	1047.38
P(3HB-co-9.9 mol.%3HV)					
30.8	25.6	788	244.00	318,71	1780.01
P(3HB-co-37.4 mol.%3HV)					
4.7	49.6	235	196.35	244.61	1038.91
P(3HB-co-11.9 mol.%4HB)					
3.7	20.8	77	111.94	141.98	605.22
P(3HB-co-21.1 mol.%4HB)					
1.7	103.2	173	131.20	167.60	641.01

## Data Availability

Not applicable.
